# Revisiting congenital rubella data in Brazil: caution in interpreting administrative records

**DOI:** 10.1017/ash.2026.10352

**Published:** 2026-03-27

**Authors:** Camila Ohomoto Morais, Daniel Jarovsky, Flavia Jacqueline Almeida, Marco Aurelio Palazzi Safadi

**Affiliations:** Pediatrics, Santa Casa de Sao Paulo School of Medical Sciences, Sao Paulo, Brazil

Dear Editor,

We read with great interest the article by Callado et al., “Hospitalizations for congenital infections in Brazil’s unified health system: nationwide trends and regional disparities, 2008–2024,” published in Antimicrobial Stewardship and Healthcare Epidemiology.^1^ The authors should be acknowledged for addressing congenital infections using nationwide SUS hospitalization data over a long-time horizon.

However, we are compelled to express serious concern regarding the manuscript’s reporting and interpretation of congenital rubella-related hospitalizations.

In the article, the authors report large numbers of hospitalizations attributed to congenital rubella (ICD-10 P35.0), including 4,093 admissions cumulatively between 2010 and 2024.^1^ These Figures are not only unexpected but appear fundamentally incompatible with Brazil’s epidemiological reality and with the established elimination status of rubella and congenital rubella syndrome (CRS) in the Region of the Americas.^2,3^

**Figure 1.**
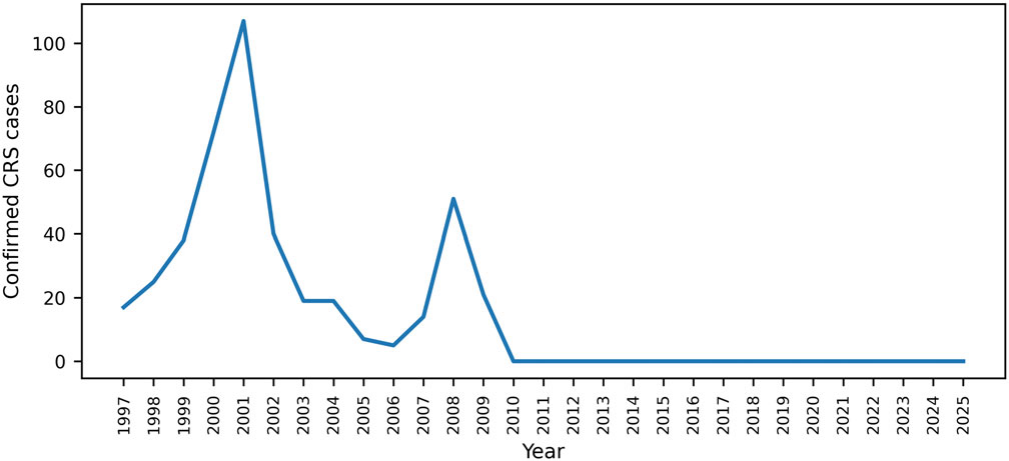
Confirmed cases of congenital rubella syndrome (CRS) in Brazil, 1997–2025.

Brazil achieved elimination of endemic rubella and CRS following sustained high vaccination coverage and strengthened surveillance efforts. After implementation of nationwide immunization strategies, including the 2008 mass vaccination campaign that targeted adolescents and adults up to 39 years of age, with coverage exceeding 95%, rubella transmission declined sharply. According to official Ministry of Health surveillance data, confirmed CRS cases peaked in the early 2000s and during the 2008 outbreak but fell to 21 cases in 2009, with zero confirmed cases reported annually from 2010 through 2025 (Figure 1).

This sustained absence of confirmed CRS cases over more than a decade demonstrates interruption of endemic transmission and maintenance of elimination status. The successful elimination of rubella and CRS in Brazil represents a major public health achievement, reflecting the effectiveness of the National Immunization Program, high MMR coverage, and continuous epidemiologic surveillance capable of detecting even isolated or imported cases.^2–4^

In this context, the persistent reporting of hundreds of congenital rubella hospitalizations per year strongly suggests that the SIH/SUS (Hospital Information System/Unified Health System) coding used in the study is capturing misclassified diagnoses rather than true CRS cases. The manuscript does not sufficiently acknowledge this likelihood and instead presents the data in a manner that may inadvertently imply ongoing rubella circulation or sustained CRS burden in Brazil.

Moreover, the article states that “rubella declined following immunization” and that rubella “remained low,” yet the hospitalization counts shown remain substantial and persistent over time, including oscillations after 2018.

Even if declining, the absolute numbers presented would represent a major and alarming public health failure, one that would almost certainly have been detected and documented by national surveillance systems. The absence of such surveillance evidence makes the study’s rubella findings highly questionable.

The authors also state that SIH case notification “integrates laboratory evidence (eg, serology or pathogen detection), compatible clinical findings in the newborn, and maternal epidemiological history.” However, SIH is primarily an administrative database designed for reimbursement and is well known to be vulnerable to diagnostic miscoding, particularly for rare congenital infections. Without validation against surveillance systems (eg, Notifiable Diseases Information System - SINAN) or laboratory-confirmed CRS registries, the interpretation of ICD-10 P35.0 as representing actual CRS cases is scientifically unsafe.

In this regard, current Brazilian Ministry of Health guidance explicitly discourages IgM testing for measles and rubella outside established clinical and epidemiologic criteria, emphasizing that isolated IgM-reactive results, particularly in individuals who do not meet the suspected case definition, may represent false positives and should not be interpreted as confirmed cases.^5^

Importantly, the dissemination of these rubella estimates without adequate qualification may have significant concerning consequences: it risks generating misinformation about rubella epidemiology, undermining the credibility of elimination certification, and potentially distorting public health priorities.

We strongly recommend that the authors revise the manuscript to explicitly address the high probability of misclassification of rubella hospitalizations in SIH/SUS, and that they avoid presenting these data as reflecting true CRS burden. At a minimum, the manuscript should include a clear warning that the rubella findings are inconsistent with Brazil’s elimination status and may reflect limitations of administrative coding. Ideally, rubella-related admissions should be validated against surveillance-confirmed CRS data or excluded from causal interpretation.

We believe this clarification is essential to ensure accurate interpretation and to prevent misleading conclusions regarding congenital rubella in Brazil.

Sincerely,
